# Macropinocytosis is responsible for the uptake of pathogenic and non-pathogenic mycobacteria by B lymphocytes (Raji cells)

**DOI:** 10.1186/1471-2180-12-246

**Published:** 2012-10-31

**Authors:** Blanca Estela García-Pérez, Juan José De la Cruz-López, Jorge Ismael Castañeda-Sánchez, Ana Rosa Muñóz-Duarte, Alma Delia Hernández-Pérez, Hilda Villegas-Castrejón, Ethel García-Latorre, Angel Caamal-Ley, Julieta Luna-Herrera

**Affiliations:** 1 Immunology Department, Escuela Nacional de Ciencias Biológicas, Instituto Politécnico Nacional, Prolongación de Carpio y Plan de Ayala s/n, Z.P. 11340, México, D.F, México; 2 Electron Microscopy Laboratory, Instituto Nacional de Rehabilitación, Av. México-Xochimilco No. 289, Col. El Arenal de Guadalupe, Tlalpan, México, D.F, México

**Keywords:** Macropinocytosis, B lymphocytes, Raji cells, *Mycobacterium smegmatis*, *Mycobacterium tuberculosis*, *Salmonella typhimurium*, Cytoskeleton

## Abstract

**Background:**

The classical roles of B cells include the production of antibodies and cytokines and the generation of immunological memory, these being key factors in the adaptive immune response. However, their role in innate immunity is currently being recognised. Traditionally, B cells have been considered non-phagocytic cells; therefore, the uptake of bacteria by B cells is not extensively documented. In this study, we analysed some of the features of non-specific bacterial uptake by B lymphocytes from the Raji cell line. In our model, B cells were infected with *Mycobacterium tuberculosis* (MTB), *Mycobacterium smegmatis* (MSM), and *Salmonella typhimurium* (ST).

**Results:**

Our observations revealed that the Raji B cells were readily infected by the three bacteria that were studied. All of the infections induced changes in the cellular membrane during bacterial internalisation. *M. smegmatis* and *S. typhimurium* were able to induce important membrane changes that were characterised by abundant filopodia and lamellipodia formation. These membrane changes were driven by actin cytoskeletal rearrangements. The intracellular growth of these bacteria was also controlled by B cells. *M. tuberculosis* infection also induced actin rearrangement-driven membrane changes; however, the B cells were not able to control this infection. The phorbol 12-myristate 13-acetate (PMA) treatment of B cells induced filopodia and lamellipodia formation, the production of spacious vacuoles (macropinosomes), and the fluid-phase uptake that is characteristic of macropinocytosis. *S. typhimurium* infection induced the highest fluid-phase uptake, although both mycobacteria also induced fluid uptake. A macropinocytosis inhibitor such as amiloride was used and abolished the bacterial uptake and the fluid-phase uptake that is triggered during the bacterial infection.

**Conclusions:**

Raji B cells can internalise *S. typhimurium* and mycobacteria through an active process, such as macropinocytosis, although the resolution of the infection depends on factors that are inherent in the virulence of each pathogen.

## Background

B lymphocytes, in addition to their role as precursors of antibody producer cells (plasma cells), are responsible for the production of cytokines such as Interleukin (IL)-4, IL-6, IL-10, and tumour necrosis factor-alpha (TNF-α) [1] and act as antigen-presenting cells; consequently, these cells are essential in the adaptive immune response. Most antigen-presenting cells, such as macrophages and dendritic cells, take up antigens in bulk to sense the extracellular environment. B lymphocytes, however, recognise specific antigens in soluble or membrane-bound forms through the B-cell receptor (BCR) [2]. Upon BCR interaction with the antigen, a cascade of signal transduction and second messengers is generated and the antigen is internalised and subsequently processed for presentation through the major histocompatibility complex (MHC) II molecules for recognition by T cells [3]. The internalisation of the antigen in B cells occurs through at least two endocytic pathways: clathrin-mediated endocytosis and clathrin- and caveolin-independent endocytosis [3,4]. However, B cells also express a number of membrane receptors that initiate the innate immune response. These receptors include the Toll-like receptors (TLR) 1, 2 (low), 4 (low), and 6, 7, 9, and 10 [5]; low levels of DEC-205, which is a putative antigen uptake processing receptor [6]; the scavenger receptor Cluster of Differentiation 36 (CD36) [7]; and the Dendritic Cell-Specific Intracellular adhesive molecule-3-Grabbing Nonintegrin (DC-SIGN) [8]. Of these, DC-SIGN is present only after B-cell activation by CD40L and Interleukin (IL)-4, which makes the B-cell able to internalise Human Herpes virus 8 (HHV 8) [8,9]. In addition, CD36 enhances Toll-like receptor 2 (TLR2) signalling to induce cytokine production [7]. In contrast to the internalisation and procession of soluble antigen, the internalisation of particulate antigen by B cells has not been extensively investigated because, unlike “professional” phagocytes, B cells do not achieve phagocytosis [10]. However, recent evidence has shown that B cells can handle and process particle antigens, bacteria, and even protozoan parasites [10,11]. It has been demonstrated that the particulate presentation of a BCR-recognised soluble antigen enhances the adaptive response by up to 10^5^-fold [12]. In fact, the build-up of lipid-antigen in a particle can also be internalised by B cells and presented through their CD1d molecules to natural killer T (NKT) cells to induce a potent adaptive response, and this is only possible if the lipid particles are recognised by the BCR [13]. There are few studies on the uptake of bacteria by B cells. A number of bacteria, including mycobacteria [14], *Salmonella typhimurium* (ST) [15], IgM-opsonised *Staphylococcus aureus*[16], *Listeria monocytogenes*[17], and, more recently, *Francisella tularensis*[11], have been found to be internalised by B-cell lines or primary culture, although the precise mechanism that is responsible for their internalisation has not yet been elucidated. The B-cell bacterial endocytic activity has recently been recognised in lower-vertebrate species, such as fishes or frogs, and interestingly, these cells also exert potent antimicrobial activity [10]. We previously demonstrated that non-phagocytic cells, such as type II pneumocytes (A549 cells), internalised pathogenic and non-pathogenic mycobacteria through macropinocytosis [18,19], and that this process was driven by metabolically active mycobacteria (live). To extend the study on the mycobacteria-triggered endocytic pathway that is responsible for the internalisation of invading non-phagocytic cells, we decided to analyse the internalisation of *Mycobacterium tuberculosis* (MTB) and *Mycobacterium smegmatis* (MSM) in B cells using scanning and transmission electron microscopy, confocal microscopy, and endocytic inhibitors to demonstrate that in Raji B cells, both of these mycobacteria are internalised through macropinocytosis. For validation, we compared our results with the internalisation features of *Salmonella typhimurium,* which was recently described to be internalised through macropinocytosis [20].

## Methods

### B cells

The Raji cell line, a human B lymphoblast cell line, was obtained from the American Type Culture Collection (ATCC, CCL-86). The cells were grown in RPMI-1640 with 10% fetal bovine serum (FBS) and antibiotics (25 mg/L gentamicin and 50,000 U/L penicillin) at 37°C in an atmosphere with 5% CO_2_.

### Bacteria and bacterial growth supernatants

*M. tuberculosis* H37Rv (ATCC) and *M. smegmatis* mc^2^ were grown in Middlebrook 7H9 broth, which was enriched with additional OADC for the growth of *M. tuberculosis*. *Salmonella enterica* serovar Typhimurium (*Salmonella typhimurium,* ST) (ATCC 14028) was grown in Luria broth. All bacteria were cultured at 37°C until achieving log-phase growth. Immediately prior to the use of the bacterial cultures in the different experiments, one aliquot of each culture was centrifuged at 10,000 rpm. The supernatant was then collected and all remaining bacteria were removed by filtration of the supernatant through 0.22-μm filters; the bacteria-free supernatants were then maintained at −70°C until use. The bacterial pellet was suspended in Hanks’ balanced salt solution (HBSS) without phenol red and centrifuged; this washing step was repeated twice. The bacterial pellet was then resuspended in HBSS, adjusted to a McFarland number 1 tube, and diluted in RPMI-1640 medium with 1% FBS serum in the absence of antibiotics to reach the necessary bacteria-to-cell ratio.

### Survival of intracellular bacteria

A suspension of B cells adjusted to a concentration of 2 × 10^6^ cells/mL was prepared as described previously. The cells were infected with each bacterial suspension (*M. tuberculosis*, *M. smegmatis*, and *S. typhimurium*) and maintained at 37°C in a CO_2_ atmosphere. After 2 h, the non-internalised bacteria were removed by low speed centrifugation (1,000 rpm for 5 min), the supernatant was discarded, and the cells were suspended in HBSS. After this procedure was repeated three times, the cellular pellet was suspended in RPMI-1640 with 1% FBS, and 20 μg/mL of amikacin (Sigma); after two h, the concentration of amikacin was decreased to 10 μg/mL to eliminate any extracellular bacteria; in the latter medium, the cells were incubated for 12, 24, 48, and 72 h after infection with *M. smegmatis* and *M. tuberculosis* and for 6, 12, 18, and 24 h after infection with *S. typhimurium*. After each time point, the cells were washed three times with HBSS using low-speed centrifugation (1,000 rpm). To determine the number of intracellular bacteria, the washed cell pellet was lysed and resuspended in 500 μL of sodium dodecyl sulphate (SDS) (0.25%); after 3 min, 500 μL of 5% bovine serum albumin (BSA) was added. The cell lysates were collected and maintained frozen at −70°C. To determine the colony-forming units (CFU), serial dilutions of the samples that were infected with *M. tuberculosis* and *M. smegmatis* were plated on Middlebrook 7H11 agar; similarly, the serial dilutions of the samples infected with *S. typhimurium* were plated on Luria agar.

### Bacterial and fluid-phase uptake by B cells

An aliquot of B cells in log-phase growth was centrifuged at 1,000 rpm and washed three times with HBSS. After the cell viability was determined using trypan blue dye, the suspension was adjusted to a concentration of 2 ×10^6^ cells/mL in RPMI-1640 with 1% FBS and 0.1 mg/mL dextran-FITC 70 (Sigma). The set of experiments on fluid-phase uptake were settled under the following conditions: (a) 1.0 μg/mL phorbol 12-myristate 13-acetate (PMA) (Sigma), (b) bacterial supernatant diluted by 1:10 in RPMI-1640, (c) *M. smegmatis* at a multiplicity of infection (MOI) of 10:1 and (d) *M. tuberculosis* at an MOI of 10:1, (e) *S. typhimurium* at an MOI of 20:1, and (f) control medium. In a 96-well sterile culture plate, a total of 200,000 treated cells were seeded in each well. The following procedure was followed for each condition: (1) quadruplicate samples were settled; (2) the plate was incubated at 37°C in a CO_2_ atmosphere; (3) after 15, 60, 90, 120, and 180 min, the fluid-phase excess was removed by centrifugation; (4) the cells were washed three times with HBSS; and (5) the washed cells were resuspended in 100 μL of HBSS. The fluorescence at each time point was measured as relative fluorescence units (RFU) using a plate fluorometer (Fluoroskan Ascent FL, Thermo) at a 485 nm excitation and a 538 nm emission. The inhibition of the fluid-phase uptake was analysed in the presence of several inhibitors, including (a) 3 μM amiloride (AMIL), which is an ion exchange inhibitor that is used as an inhibitor of macropinocytosis [21,22], (b) 0.1 μM wortmannin (WORT), a PI3K inhibitor [23] and (c) 3 μM cytochalasin D (CD), a known inhibitor of actin polymerisation [24]. All of the inhibitors were purchased from Sigma. Each inhibitor was added to the respective cellular suspensions 30 min prior to treatment and was not removed during the experiment. The cells were processed as previously mentioned, and the resultant RFUs were recorded. The B-cell line viability in the presence of these inhibitors was monitored during the experiment. The cell viability was assessed by staining an aliquot with 0.2% trypan blue and calculating the percentage of cells that were not dyed. The viability in the control (no inhibitor) and treated cells reached 95%. The fluid-phase uptake data were analysed for statistical significance using one-way analysis of variance (ANOVA) using the SigmaStat software*. P* values ≤ 0.01 were considered statistically significant. The inhibition of the bacterial uptake was also analysed in the presence of amiloride using a protocol similar to that used in the previous experiments. Concentrations of 1, 3 and 5 mM of amiloride were added to the cells 30 min prior to the addition of the bacteria; the inhibitor was maintained in the samples throughout the 90 min during which the bacterial uptake occurred. A set of untreated cells were infected with the same bacterial suspension for control. At the end of the incubation, the extracellular bacteria were removed by centrifugation, and the CFUs were determined as described previously. The cell viability was also assessed at the end of the experiment and was found to reach >90% regardless of the concentration of inhibitor that was used.

### Transmission electron microscopy (TEM)

Some of the features of the infection of B cells with *M. tuberculosis*, *M. smegmatis*, and *S. typhimurium* were analysed by TEM. Because PMA is known to act as a macropinocytosis inducer [25], the features of B cells under PMA treatment were also analysed. B-cell suspensions were treated with 1.0 μg/mL of PMA for 1 h or infected for 1 and 24 h with the following bacterial suspensions: *M. tuberculosis* at an MOI of 10:1; *M. smegmatis* at an MOI of 10:1, and *S. typhimurium* at an MOI of 20:1. After treatment and infection, the suspension cells were washed four times by centrifugation at 1,000 rpm with PBS solution to remove any non-internalised bacteria and excess PMA. The cells were fixed with 2% glutaraldehyde solution in 0.1 M PBS for 2 h at room temperature. The cells were then washed three times with PBS and post-fixed with osmium tetroxide for 1 h at 4°C. The osmium tetroxide was removed by centrifugation with PBS, and the pellet was processed following the standard procedure for TEM analysis [18]. The final sections obtained were examined under a transmission electron microscope (Philips, Tecnai 10, Holland).

### Scanning electron microscopy

Fresh B-cell suspensions were prepared (1 × 10^6^ cells/mL) and infected with non-labelled *M. smegmatis*, *M. tuberculosis*, or *S. typhimurium* for 1 h at 37°C and 5% CO_2_, according to the protocol described previously; in addition, some B-cell suspensions were treated with PMA instead of the bacterial cultures. The non-internalised bacteria or the excess PMA was removed by centrifugation using PBS, as described previously; the cell pellet was then fixed with 2% glutaraldehyde solution in PBS for 2 h at room temperature. The cells were then washed three times with PBS, post-fixed with osmium tetroxide for 1 h at 4°C, and processed as previously described [18]. The cells were observed using a scanning electron microscope (Jeol-JSM-5800LV, Japan).

### Fluorescein isothiocyanate (FITC) bacterial staining

To analyse the cytoskeletal rearrangements and bacterial intracellular localisation by confocal microscopy, the *M. smegmatis*, *M. tuberculosis*, and *S. typhimurium* bacteria were stained with Fluorescein isothiocyanate (FITC) (Sigma). The staining protocol included the following steps: (1) 1 mL of a McFarland number 3 bacterial suspension was washed by centrifugation, (2) the bacterial pellet was suspended in 1 mL of a phosphate buffered saline (PBS) solution (0.15 M, pH 7.2) that contained 0.1 mg/mL of FITC, and (3) the bacterial suspension was incubate for 30 min at 37°C. The remaining dye was removed by centrifugation with PBS until the supernatant did not register any fluorescence when read on a plate fluorometer at a 485 nm excitation and a 538 nm emission (Fluoroskan Ascent FL, Thermo). The dyed bacterial pellet was adjusted to a McFarland number 1 tube in HBSS and then utilised in the respective experiments.

### Confocal microscopy

A suspension of B cells at a concentration of 1 × 10^6^ cells/mL was processed as mentioned previously. The cells in suspension were infected for 1 and 3 h using a bacterial suspension of FITC-labelled *M. tuberculosis*, *M. smegmatis*, or *S. typhimurium*. The infections were performed at 37°C in an atmosphere with 5% CO_2_. Following infection, the non-internalised bacteria were removed through five rounds of centrifugation at low speed (1,000 rpm) and using HBSS for the resuspension of the B cells after each centrifugation. The cells were then fixed with 4% paraformaldehyde for 1 h at room temperature. A cell monolayer was then formed on a glass slide in a Cytospin 3 (Thermo) through the centrifugation of the fixed cells at 700 rpm for 5 min. The monolayer was washed twice with PBS and the cells were permeabilised for 10 min with a 0.1% Triton X-100 solution in PBS. The cells were then washed twice with PBS and covered with 80 ng of a rhodamine-phalloidin solution (Sigma) per cover slip for 20 min at room temperature. Excess phalloidin was removed by washing five times with PBS. The labelled preparations were mounted on a glass slide with Vectashield solution (Vector Laboratories) and observed using a confocal laser scanning microscope system attached to a microscope (LSM 510, Zeiss).

## Results

### Survival of intracellular bacteria

**Figure 1:**
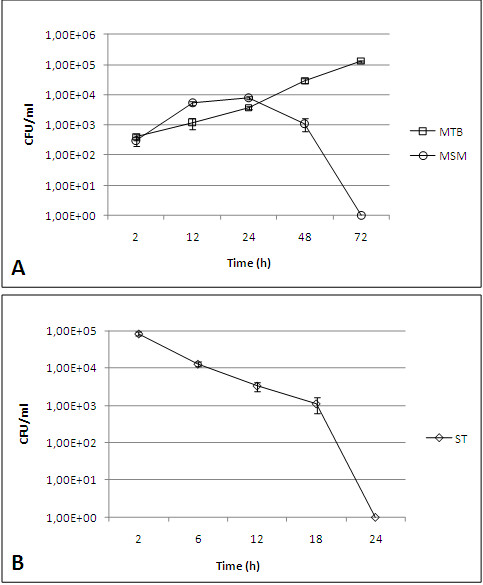
**Colony forming units (CFU) of *S. typhimurium *and mycobacteria in B cells. a**) Time-dependent CFU counts of intracellular *M. smegmatis *(MSM) (circles) and *M. tuberculosis* (MTB) (squares). The growth of *M. smegmatis *is controlled by the end of the kinetics, whereas *M. tuberculosis *survives and multiplies. **b**) Time-dependent CFU counts of intracellular *S. typhimurium *(ST). The intracellular growth was rapidly controlled by the B cells compared to the mycobacteria. Each point represents the mean ± standard error (SE) of triplicate measurements. The experiment presented is representative of three independent repetitions.

To determine whether mycobacteria can replicate in B cells, antibiotic-protection assays were conducted. The *S. typhimurium* bacteria were completely eliminated by B cells (Figure 1b); in addition, although *M. smegmatis* underwent brief replication during the first 24 h of infection, an important decrease in the intracellular bacteria was observed starting at 48 h and through the end of the post-infection kinetics (Figure 1a). *S. typhimurium* did not present any intracellular replication; in fact, at 6 h post-infection (Figure 1b), a significant decrease in the bacterial load was observed, which resulted in total bacterial elimination. In contrast, the internalised *M. tuberculosis* exhibited intracellular growth in B cells and sustained exponential growth throughout the experiment (72 h after infection) (Figure 1a).

### Fluid-phase uptake by infected B cells

**Figure 2:**
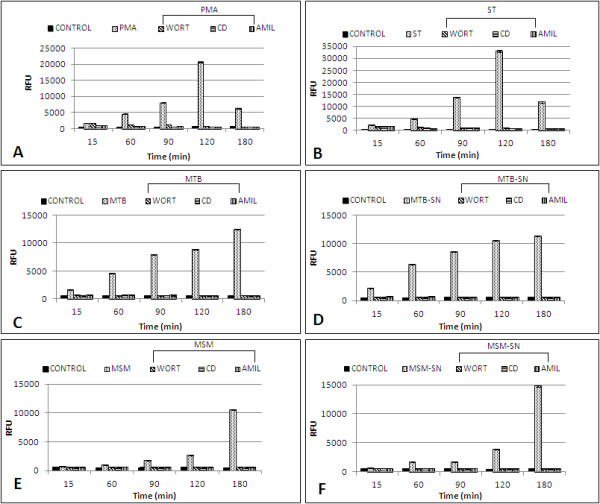
**Fluid-phase uptake by Raji B cells induced by different treatments. **B cells were infected with *M. tuberculosis *(MTB), *M. smegmatis *(MSM), and *S. typhimurium* (ST), or treated with phorbol 12-myristate 3-acetate (PMA), *M. tuberculosis *culture supernatant (MTB-SN), or *M. smegmatis *culture supernatant (MSM-SN). The fluorescent fluid-phase uptake was determined by the quantification of the relative fluorescence units (RFU) at several time points (15, 60, 90, 120, and 180 min). B cells that were not treated served as the control (CONTROL) for each treatment. The effect of several inhibitors on the fluid-phase uptake was also monitored. Each of the inhibitors (cytochalasin (CD), wortmannin (WORT), and amiloride (AMIL) was individually added to the following treatments/infections: **a**) PMA treatment, **b**) ST, **c**) MTB, **d**) MTB-SN, **e**) MSM, **f**) MSM-SN. Each bar represents the mean of four different measurements. There were statistically significant differences (*p *<0.01) when the infected, PMA-treated and SN-treated B cells were compared with i) the control cells, ii) the infected cells in the presence of the inhibitors, and iii) the PMA-treated or SN-treated cells in the presence of the inhibitors. The experiment presented is representative of three independent repetitions.

Untreated (control) B cells exhibited a very low capability for fluid-phase uptake (Figure 2a-f); however, these cells presented an RFU time- and treatment-dependent increase in fluid-phase uptake under several experimental conditions. The *S. typhimurium* infection induced the highest fluid-phase uptake, with a peak reached after 120 min of infection, but the RFU values were found to decrease thereafter (Figure 2b). *M. tuberculosis* induced a sustained RFU increase (Figure 2c), but the RFU values were lower than those achieved with *S. typhimurium*. *M. smegmatis* triggered the lowest and slowest uptake (Figure 2e). Furthermore, PMA was the best inducer of fluid-phase uptake, but the RFU values were not as high as those reached with *S. typhimurium*. Similar to the kinetics observed with *S. typhimurium*, after the RFU peak was reached, a decrease in the fluorescence was observed for PMA (Figure 2a). The mycobacterial supernatants induced uptake tendencies that were similar to those observed with their respective bacteria (MTB-SN induced the highest and fastest uptake) (Figures 2d and 2f). Interestingly, only live bacteria (*S. typhimurium*, *M. tuberculosis*, and *M. smegmatis*) triggered this phenomenon because heat-treated bacteria did not induce any fluid-phase uptake (data not shown).

### Effect of inhibitors on bacterial and fluid-phase uptake by B cells

**Figure 3:**
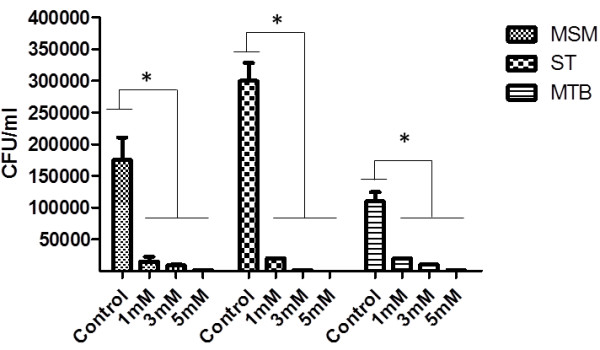
**Bacterial uptake by Raji B cells is inhibited by amiloride treatment. **B cells were infected with *M. tuberculosis *(MTB), *M. smegmatis *(MSM), and *S. typhimurium *(ST) for 90 min. The cells were treated with 1, 3 or 5 mM amiloride before and during the infection. The CFU counts were determined and recorded; * statistically significant differences (*p *<0.01) when the untreated/infected cells were compared with amiloride-treated/infected cells.

To determine the pathway responsible for the bacterial and fluid-phase uptake that was previously observed in the B cells, several classical endocytic inhibitors were employed [26], including AMIL (macropinocytosis), CD, and WORT (macropinocytosis and phagocytosis). In addition, bacterial infections and soluble treatments (PMA or mycobacterial supernatants) were used in these experiments. The fluid-phase uptake induced during bacterial infections was completely abolished by AMIL, WORT, and CD (Figures 2a through f), and this inhibition was observed throughout the experiment. Similarly, the fluid-phase intake triggered by PMA, *M. tuberculosis,* or the *M. smegmatis* supernatant was suppressed by these inhibitors (Figures 2a, 2d and 2f). The inhibition in all these cases was statistically significant. In addition, the bacterial uptake was inhibited with amiloride at all concentrations used (Figure 3). The ST and MSM uptakes were the most affected. Even at the lowest inhibitor concentration used (1 mM), a high uptake inhibition was observed with all bacteria. These observations indicated that macropinocytosis was responsible for the uptake of bacteria into these cells.

### Transmission electron microscopy of infected B cells

**Figure 4:**
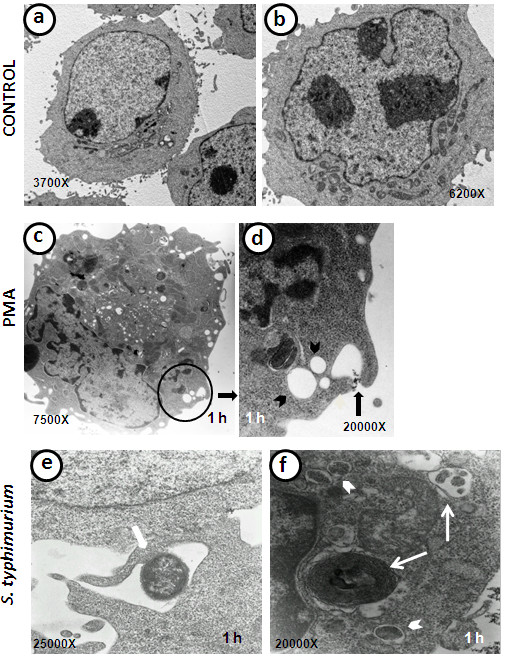
**Ultrastructure of B cells infected with *S. typhimurium *(ST) and stimulated with phorbol 12-myristate 3-acetate (PMA). a-b**) Control B cells. **c**) PMA-stimulated B cell, which has abundant vacuoles of different sizes. **d**) The field magnification of a PMA-stimulated B cell (circle) shows macropinosome formation (black narrow) and the presence of macropinosomes that are already formed in various sizes (arrowheads). **e**) Micrograph of *S. typhimurium-*infected B cell, which shows that the bacillus is surrounded by large membrane extensions (narrow). **f**) *S. typhimurium*-infected B cell with internalised bacteria (arrowheads), thin narrows depicts a multilamellar structure (left) and a late degradative autophagic vacuole (LDAV) (right).

**Figure 5:**
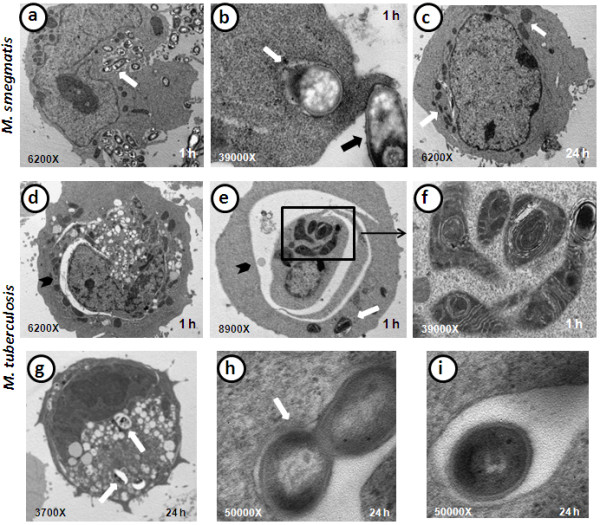
**Ultrastructure of B cells infected with *M. smegmatis *(MSM) and *M. tuberculosis *(MTB). a**) MSM-infected B cell with abundant internalised bacilli (white arrow) after 1 h of infection. **b**) MSM-infected B cell after 1 h of infection, which shows the binding of a bacillus to a lamellipodium (black arrow) and the destruction of an intracellular bacillus contained in a vacuole (white arrow). **c**) MSM-infected B cell at 24 h post-infection, which shows that the cell morphology was recovered and that no internalised bacilli were present, although some swollen mitochondria were still observed (white arrows). **d**-**e**) After 1 h of infection, a B cell infected with MTB exhibits a large number of alterations, abundant vacuoles, swollen mitochondria, internalised mycobacteria (white arrow), and “curved vacuoles” (black arrowheads). **f**) Magnification of a B cell infected with MTB (square), which shows that some of the altered mitochondria are in the process of forming double to multi-membrane vacuoles (autophagy-like vacuoles). **g**) B cell infected with MTB for 24 h shows intracellular bacilli in vacuoles (white arrows), abundant vacuoles, and an electro-dense cellular nucleus, which suggests strong damage. **h**) Replicating mycobacteria in spacious vacuole (white arrow) formed in a B cell infected with MTB for 24 h. g) Detail of MTB bacillus in a spacious vacuole after 24 h of B cell infection.

To establish the ultrastructural changes that are induced by mycobacteria, the cells were analysed using transmission electron microscopy. The uninfected cells exhibited a round shape, a low cytoplasm/nuclei ratio, and scarce and small membrane projections; therefore, no significant internalisation features were observed (Figures 4a and 4b). When the cells were infected or treated with soluble components, a number of changes were observed. The PMA-treated cells exhibited a large number of vacuoles or macropinosomes of different sizes (Figures 4c and 4d). As shown in Figure 4e, *S. typhimurium* induced the formation of membrane extensions, such as lamellipodia. In addition, intracellular bacteria were observed and were found to be surrounded by these membrane projections (Figure 4f). In some *Salmonella*-infected cells, a number of structures, such as double membrane vacuoles and multilamellar bodies, were observed (Figure 4f). *M. smegmatis* induced long membrane projections, which surrounded the bacteria (Figure 5a). Some intracellular mycobacteria were observed to have cell wall damage (Figure 5b). At 24 h post-infection, it was difficult to find any internalised bacilli, and the cellular morphology was similar to that of uninfected cells, although some large mitochondria were still observed (Figure 5c). In contrast, major ultrastructural changes due to *M. tuberculosis* infection were evident: the infected cells contained abundant vacuoles of different sizes and shapes and, in many cases, these vacuoles exhibited an extended and curved shape and were found in close proximity to the nuclei (Figure 5d). In addition, the *M. tuberculosis*-infected cells showed abundant swollen mitochondria and, frequently, mitochondria that were sequestered into double membrane structures (Figures 5e and 5f). After 24 h of infection with *M. tuberculosis*, the cells did not recover their basal morphology and still presented abundant vacuoles (Figure 5g). Unlike *M. smegmatis* and *S. typhimurium*, intracellular *M. tuberculosis* replicated well in these cells (Figures 5h) and the bacterial morphology was excellent (5i).

### Scanning electron microscopy of infected Raji B cells

**Figure 6:**
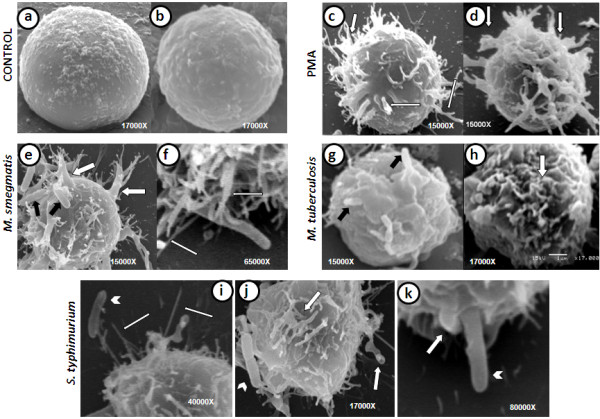
**Scanning electron micrographs of B cells infected with mycobacteria or *S. typhimurium *(ST) or treated with phorbol 12-myristate 3-acetate (PMA). a**-**b**) Non-infected B cells. **c**-**d**) PMA-treated B cells, which exhibit abundant long, thin, and wide membrane extensions that resemble filopodia (thin arrows) and lamellipodia (wide arrows). **e**-**f**) B cells infected with *M. smegmatis *(MSM) show abundant membrane filopodia (thin white narrows) and lamellipodia formation (wide white narrows) and attached bacilli that are trapped by the membrane projections (black arrows). **g**-**h**) *Mycobacterium tuberculosis *(MTB)-infected B cells show membrane ruffling (white arrow) and some bacilli bound to the cell (black arrows). **i**-**k**) *S. typhimurium*-infected B cells show filopodia (thin white arrows) and lamellipodia formation (wide white arrows). The white arrowheads depict attached bacilli and a bacillus that is surrounded by forming lamellipodia.

The resting B cells (Figures 6a and 6b) possessed a smooth to slightly irregular membrane. However, drastic changes in the membrane ultrastructure were observed with the different treatments that were administered. PMA, which is known as a classical macropinocytosis inducer, induced the formation of membrane ruffling, filopodia, and lamellipodia that entirely surrounded the cells (Figures 6c and 6d). *M. smegmatis* (Figures 6e and 6f) and *S. typhimurium* (Figures 6i, 6j and 6k) induced a similar phenomenon: membrane ruffling and filopodia formation that completely covered the cell. The bacteria were also found to be attached either to the cell by membrane ruffles (Figures 6e and 6j) or long filopodia (Figures 6j and 6i) or to inside the cell (Figure 6k). In contrast, *M. tuberculosis* infection mainly induced membrane ruffling (Figures 6g and 6h), and the bacilli were trapped by the wide membrane sheets (Figure 6g). All of these images resemble macropinocytic processes, which confirm the TEM observations, the fluid-phase results and the bacterial uptake data that were presented previously.

### Cytoskeletal role: actin filaments

**Figure 7:**
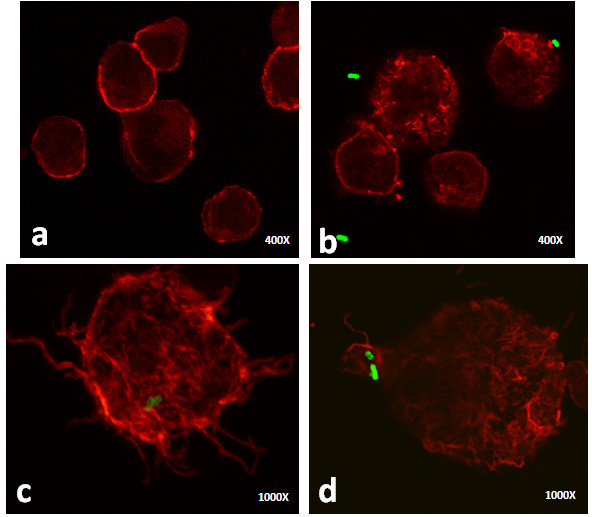
**Confocal images of uninfected and *S. typhimurium *(ST)-infected B cells. **The actin filaments were labelled with rhodamine-phalloidin and the bacteria were stained with fluorescein isothiocyanate (FITC). **a**) Uninfected cells present peripheral and homogeneous fluorescent staining. **b**) One h after infection, *S. typhimurium *induced actin cytoskeletal rearrangements that are responsible for membrane ruffling; in addition, a bacillus that is attached to these structures was observed (upper right corner). **c**-**d**) After 3 h of infection, longer actin projections and actin redistribution were observed, and some bacilli were found inside the B cell (**c**) or surrounded by actin organisations (**d**).

**Figure 8:**
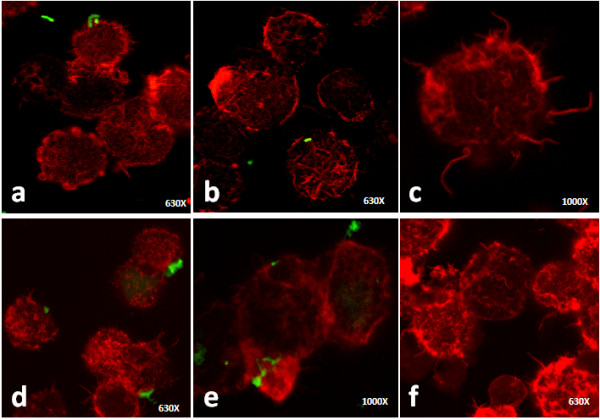
**Confocal images of B cells infected with mycobacteria. **The actin filaments were labelled with rhodamine-phalloidin and the bacteria were stained with fluorescein isothiocyanate (FITC). **a**) *M. smegmatis *(MSM) infection caused evident actin rearrangements within 1 h of infection; a mycobacterium was observed attached to the cell. **b**-**c**) After 3 h of infection with MSM, intracellular bacteria were observed (**b**) and long actin filaments were evident (**c**). **d**) *M. tuberculosis *(MTB) infection induced actin reorganisation after 1 h of infection, and bacilli attached to the cells were observed; **e**-**f**) B cells, after 3 h of infection with MTB, presented actin cytoskeletal changes in cells without any adhered or intracellular bacteria.

To establish the role of the actin filaments on the mycobacterial internalisation, we performed confocal analyses. The actin filaments were stained with phalloidin-rhodamine and the bacteria were labelled with FITC. The uninfected cells presented a peripheral fluorescent label that sustained the spatial cell morphology (Figure 7a). The *S. typhimurium*-infected cells lost the regular peripheral fluorescent label. After 1 h of infection, the actin cytoskeletal rearrangements resulting in membrane ruffling were evident on the cell surface, and the attachment of the bacilli to these structures was observed (Figure 7b). After 3 h of infection, the cells exhibited long actin projections and actin re-distribution (Figure 7c). Additionally, some bacilli were found adhered to the actin organisations that resulted in the lamellipodia formation (Figure 7d). Furthermore, these changes were also observed in cells without any adhered or internalised bacteria (Figure 7b). *M. smegmatis* infection caused actin rearrangements that could terminate in membrane ruffling, lamellipodia, and filopodia formation. Some cells also showed actin focal spots on the cell surface (Figures 8a, 8b and 8c). After 3 h of infection, long actin filaments, which are responsible for the formation of membrane filopodia, were present on the cell surface (Figure 8c). *M. smegmatis* infection-associated actin rearrangements were evident in all of the cells that were present in the preparation, although some cells did not present either adhered or internalised bacilli (Figures 8a, 8b and 8c). *M. tuberculosis* infection induced actin reorganisation that was responsible for membrane ruffling (Figures 8d, 8e and 8f), although fewer long actin filament formations were observed compared to the other infections (Figures 8d and 8f). Further, adhered and internalised bacilli were evident after 1 and 3 h of infection, respectively (Figures 8d and 8e). As with all of the other infections, all of the actin cytoskeletal changes were also evident in cells without any adhered or intracellular bacteria (Figure 8f).

## Discussion

The classical B-cell roles include the production of antibodies and cytokines and the generation of immunological memory, which are key factors in the adaptive immune response. Recently, their role in innate immunity is been increasingly recognised [27,28]. The majority of studies that analyse B-cell specific-antigen recognition mainly focus on soluble antigens. B cells have been traditionally considered non-phagocytic cells [29]; therefore, the process of bacterial uptake by B cells has not been extensively documented.

Mature B cells bind specific soluble protein antigens through a unique and restricted BCR [30-32]. At present, it is known that the Antigen-B-cell receptor (Ag-BCR) complex is internalised into clathrin-coated pits, although raft signalling and actin polymerisation are required for efficient receptor mediated-endocytosis [33]. The binding of antigens to the BCR induces cell signalling and triggers changes in the actin cytoskeletal organisation, although these changes are limited to the vicinity of the Ag-bound BCR and are not generalised throughout the cellular membrane [33,34]. The actin reorganisation after the BCR-antigen engagement is a rapid albeit transient event [34]. The internalised BCR transports the Ag to the endosomal compartments, where it is fragmented and loaded onto nascent MHC class II proteins for its presentation to T cells [31].

In contrast to BCR-mediated specific soluble-antigen uptake, we studied the features of non-specific bacterial uptake by B cells. In our study, B lymphoblast cells of the Raji cell line (Burkitt’s lymphoma) were infected with two *Mycobacterium* species and with *S. typhimurium*, and the resultant cellular membrane changes and cytoskeletal reorganisation events were analysed. We previously reported that macropinocytosis is the mechanism responsible for the internalisation of mycobacteria into the lung epithelial A549 cell line [18,19] and into endothelial cells [35]. Therefore, considering that B lymphocytes have been recognised as classical non-phagocytic cells [29], we sought to establish whether mycobacteria were able to induce macropinocytic internalisation in B cells. In our design, the infections were conducted with B cells in suspension; to avoid the spreading feature that is commonly observed in these cells, we did not plate Raji cells on any cell surface that was either uncovered or covered with any extracellular matrix ligands or antibodies [36,37].

Our observations revealed that the B cells were readily infected by the three bacteria that were studied and that the infections induced relevant changes in the cellular membrane during bacterial internalisation (Figure 6). *M. smegmatis* is considered a non-pathogenic mycobacteria; however, it was able to induce important membrane changes that were characterised by abundant filopodia and lamellipodia formation (Figure 6e and 6f) and were similar to those triggered by PMA (Figures 6c and 6d). B cells that were treated with the supernatant from the bacterial cultures (mycobacteria were removed by centrifugation and filtration) exhibited the same ultrastructural changes (data not shown). *M. smegmatis* was readily internalised; in fact, some cells internalised a large number of the mycobacteria (Figure 5a). *M. smegmatis* exhibited a transient multiplication, which was revealed by the counting of CFU 12 and 24 h post-infection (Figure 1a). However, by 48 and 72 h, the mycobacteria were eliminated. After 24 h of infection, no evident intracellular mycobacteria were observed on the TEM images, and the B cell morphology was similar to that of uninfected cells (Figure 5c). Intravacuolar mycobacteria destruction was clearly observed, and partial destruction of the bacterial cell wall was evident (Figure 5b). The results from the analysis of mycobacterial intracellular elimination, membrane protrusion formation, and cytoskeleton rearrangements during bacterial uptake resemble those observed in the infection of epithelial and endothelial cells by *M. smegmatis*[19,35], although *M. smegmatis* induced significantly fewer changes in endothelial cells. To our knowledge, there are no other reports of B cell infection by *M. smegmatis*; therefore, this study is the first description of this subject.

The *M. tuberculosis* infection of B cells showed some differences with the effect of *M. smegmatis* and *S. typhimurium* infections. *M. tuberculosis* has previously demonstrated the capability to invade several cell types, including epithelial [18,38], fibroblast [39], and endothelial cells [35,40]. The cellular membrane protrusions formed during *M. tuberculosis* internalisation have been described in some of these cells [18,35,40]. In B cells, membrane protrusions were also observed during *M. tuberculosis* uptake. However, these protrusions were different from those observed with *M. smegmatis* and *S. typhimurium* infections or with PMA treatment; these appeared to be wider and shorter compared with those formed during the other treatments/infections (Figures 6g and 6h). In addition, no evident filopodia formation was observed during *M. tuberculosis* infection, and the protrusions were more similar to ruffles. The actin cytoskeleton sustained these membrane protrusions (Figures 8e and 8f), although the actin filaments were shorter compared to those formed during PMA treatment and *M. smegmatis* or *S. typhimurium* infection. Of the three bacteria utilised for the infection of B cells, only *M. tuberculosis* was able to survive and multiply intracellularly (Figure 1). In an earlier study of *M. tuberculosis* uptake by human-transformed B cells [14], the authors described the formation of membrane protrusions during mycobacterial infection that were similar to those described by our group. The authors also demonstrated the presence of mycobacteria in spacious vacuoles and the presence of abundant mitochondria in infected cells. The authors indicated that the internalisation of live *M. tuberculosis* by B cells results in the presentation of the mycobacterial antigen to T cells. A number of characteristic structures were observed in B cells that were infected with *M. tuberculosis*, including “curved vacuoles” with arched or crescent shapes (Figures 5d and 5e), which contain amorphous material. Because these structures were not observed with the other infections, they appear to be characteristic of *M. tuberculosis* infection.

In our study, we were unable to observe *Salmonella-*induced filaments (SIFs), which are the hallmark organelles in which the bacteria multiply in epithelial cells [41,42]. This observation might be the result of the rapid elimination of *Salmonella* from the B cells. To our knowledge, there is currently no description of SIF formation in *Salmonella*-infected B cells.

B-cell infection by *S. typhimurium* has been previously reported [29,43,44]. It is known that *S. typhimurium* is internalised through macropinocytosis in several cell models, such as epithelial cells and macrophages [45,46]. It was recently demonstrated that *S. typhimurium* can infect B cells by macropinocytosis [20]. Thus, we utilised the *Salmonella* infection of B cells as a positive control to corroborate that the process induced during mycobacterium internalisation by B cells was macropinocytosis. All of the features observed during B cell infection by *Salmonella* were consistent with the phenomenon of macropinocytosis, including the membrane protrusion formation (Figure 6j), actin involvement (Figures 7b, 7c and 7d), and spacious vacuole formation (Figure 4e and 4f) [46-48]. Therefore, due the morphological evidence and the inhibition of bacterial internalisation by amiloride, we can conclude that *S. typhimurium* induced macropinocytosis for its internalisation into the Raji B cell, which confirms the recent findings on the internalisation of *S. typhimurium* into mouse primary B cells [20]. During macropinocytosis, large volumes of fluid-phase are found in spacious vacuoles known as macropinosomes; therefore, the quantification of the fluorescent-labelled fluid-phase could be an indirect measurement of the macropinocytosis that is triggered by some inducer [49,50]. In this context, *S. typhimurium* induced the highest uptake by B cells. The level of internalisation of *S. typhimurium* was higher than that achieved with PMA, which is considered an efficient inducer of macropinocytosis [25]. Both of the mycobacteria induced a lower uptake; however, in contrast to *Salmonella* or PMA, we did not observe any reduction in the fluorescence uptake throughout the experiment. The use of pharmacological inhibitors complements the study of endocytosis and aids in the elucidation of the endocytic processes that occur in different cells [26,49,50]. In this study, we found that, during *Salmonella* or mycobacteria infections, the fluid-phase uptake was abolished by CD, WORT, and AMIL, confirms the involvement of the cytoskeleton during the infection, the participation of PI-3K, and the phenomenon of macropinocytosis as the process that is responsible for the bacterial internalisation. Interestingly, the *M. tuberculosis* and *M. smegmatis* culture supernatants (obtained during the log-phase growth of the bacteria) were able to induce the same level of fluid-phase uptake as the live bacteria. Furthermore, the supernatant fluid-phase uptake was inhibited by all of the inhibitors, which suggests that the soluble factors that are produced by these bacteria are able to induce macropinocytosis and is consistent with previous studies that have suggested this phenomenon in other cell types [18,19].

Different from other B-cell models [29,43,44], *S. typhimurium* was eliminated by the Raji B cells (Figure 1b), no replicating intracellular bacteria were observed in the *Salmonella-*containing vacuoles of these B cells, and no SIF structures were induced in the cells during the *Salmonella* productive infection [41,42]. Instead, we observed (Figure 4f) non-replicating bacteria, some of which were in the process of being destroyed, multilamellar bodies, and some late degradative autophagic vacuoles (LDAV) [51]; the presence of these structures suggests that autophagy was in progress, which could be partly responsible for the containment of the *Salmonella* growth [52], although this observation should be analysed in more detail. In contrast to the Raji B-cell line, the Ramos B-cell line can internalise only *Salmonella* that is bound to the specific anti-*Salmonella* antibody; thus, the BCR-mediated internalisation in these cells allowed Ag presentation, IgM anti-*Salmonella* production, and *Salmonella* intracellular survival [29].

B cells from early vertebrates, such as teleost fish, are able to internalise bacteria and exert microbicidal abilities [10]. In this study, Raji B cells, like the B cells from early vertebrates, were able to control *S. typhimurium* and *M. smegmatis* but not *M. tuberculosis*. The precise antimicrobial mechanisms that are exerted by B cells from cell lines or primary cells are not yet well known. To date, among the possible antimicrobial mechanisms, nitric oxide (NO) is believed to be responsible for the control of pathogen growth by B cells. The B1 subset of B lymphocytes constitutively expresses the mRNA of inducible nitric oxide synthase (iNOS) and produces NO prior to and during *Cryptococcus neoformans* infection, which contributes to the elimination of the pathogen [53]. The B1 cells also produce NO under TLR stimulation, which suggests that these cells have a role in non-specific, cell-mediated immunity against pathogens [54]. Novel recent evidence suggests that B cells may also produce defensins in response to TLR stimulation. For example, the stimulation of B cells with CpG-DNA induces the production of β-defensin 2 [55]. The scarcity of evidence on the B cell mechanisms that are involved in the destruction of pathogens and on the precise role of B cells in the innate and specific response against mycobacterial infection makes this an interesting field of research.

## Conclusions

In this manuscript, we describe the events that occurred during the internalisation of three different bacteria into a B lymphoblast cell line (Raji cell line). *M. smegmatis*, *M. tuberculosis* and *S. typhimurium* were readily internalised by Raji B cells as early as 1 h post-infection, and their uptake was inhibited in the presence of amiloride. During mycobacteria and *Salmonella* uptake, the B cells formed lamellipodia, ruffling and filopodia. After uptake, many spacious vacuoles or macropinosomes of different sizes were observed. The fluid-phase uptake that occurs during *Salmonella* or mycobacteria internalisation was abolished by amiloride, cytochalasin D or wortmannin, which confirms the involvement of the cytoskeleton during the internalisation, the participation of PI-3K, and the triggering of macropinocytosis during bacterial uptake. Death mycobacteria did not induce fluid-phase uptake in B cells. The secreted products in a *M. tuberculosis* and *M. smegmatis* culture were able to induce the same level of fluid-phase uptake as the live bacteria, and the supernatant-induced fluid-phase uptake was inhibited by all of the inhibitors, which indicates that the soluble factors that are produced by these bacteria are able to induce macropinocytosis. The B cell cytoskeleton underwent crucial rearrangements during bacterial internalisation, which signifies that the cytoskeleton plays a role during macropinocytosis. *M. smegmatis* and S*. typhimurium* were eliminated by the Raji B cells; however, *M. tuberculosis* was able to survive and multiply in these cells, which suggests that the induction of macropinocytosis does not warrant bacterial elimination or survival.

## Abbreviations

(MTB): *Mycobacterium tuberculosis*; (MSM): *Mycobacterium smegmatis*; (ST): *Salmonella typhimurium*; (AMIL): Amiloride; (WORT): Wortmannin; (CD): Cytochalasin D; (SN): Culture supernatant; (SIFs): *Salmonella*-induced filaments; (RFU): Relative fluorescent units; (CFUs): Colony forming units.

## Competing interests

The authors of this study have no conflicts of interest to report.

## Authors’ contributions

BEGP, JJDCL, JICS, ARMD and ACL carried out the experiments and prepared the samples for electron microscopy observation. ADHP and HVC processed and analysed the TEM samples. EGL participated in the design of the study and contributed to the draft and review of the manuscript. BEGP helped draft the manuscript and edited the figures. JLH conceived the study, participated in its design and coordination and coordinated the drafting of the manuscript. All authors read and approved the final manuscript.
